# Letrozole+ GnRH antagonist stimulation protocol in poor ovarian responders undergoing intracytoplasmic sperm injection cycles: An RCT

**Published:** 2017-02

**Authors:** Mahbod Ebrahimi, Firouzeh Akbari-Asbagh, Mojgan Ghalandar-Attar

**Affiliations:** *Department of Obstetrics and Gynecology, IVF Unit, Yas Hospital, Tehran University of Medical Sciences, Tehran, Iran.*

**Keywords:** GnRH-antagonist, Intracytoplasmic sperm injections, Letrozole, Ovarian reserves

## Abstract

**Background:**

Gonadotropin-releasing hormone (GnRH) antagonist protocol has been proposed as a potentially proper option for the patients with limited ovarian reserve. Nevertheless, there is no significant difference in terms of clinical pregnancy between the GnRH antagonist and agonist cycles. The use of aromatase inhibitors such as letrozole was suggested by some studies.

**Objective:**

The object of this study was to evaluate the efficacy of letrozole co-treatment with GnRH-antagonist protocol in ovarian stimulation of poor responder patients undergoing intracytoplasmic sperm injection.

**Materials and Methods:**

A double-blinded randomized control trial was conducted on 70 infertile women with poor ovarian response based on Bologna criteria in two groups: letrozole+GnRH-antagonist (LA) group and placebo+GnRH-antagonist (PA) group (n=35/each). The LA group involved at letrozole 2.5 mg daily over 5 days and recombinant human follicle stimulating hormone 225 IU/daily. The PA group received placebo over 5 days and recombinant human follicle stimulating hormone at the same starting day and dose, similar to LA group. GnRH-antagonist was introduced once one or more follicle reached ≥14 mm. The main outcome measures were the number of oocytes retrieved, fertilization rate, implantation rate, cycle cancellation rate, and clinical pregnancy rate.

**Results:**

There were no significant differences in demographic characteristics between groups. There were no significant differences between groups regarding the number of oocytes retrieved (p=0.81), number of embryos transferred (p=0.82), fertilization rate (p=0.225), implantation rate (p=0.72), total cycle cancelation rate (p=0.08), and clinical pregnancy rate (p=0.12).

**Conclusion:**

The use of letrozole in GnRH-antagonist cycles does not improve clinical outcomes in poor responder patients undergoing intracytoplasmic sperm injection.

## Introduction

Poor ovarian response (POR) to stimulation has been defined as an unsatisfactory response to adequate ovarian stimulation (1). The inadequate response can be considered as low peak estradiol (E_2_) levels, a reduced number of mature follicles, the retrieval of few oocytes, and cancellation of previous in vitro fertilization (IVF) cycle despite adequate gonadotropin stimulation (2, 3). Advance maternal age, a high follicle stimulating hormone (1) level in early follicular phase, low antral follicle count (AFC), low inhibin B, and a low anti-müllerian hormone (AMH) level determine the possibility of POR after superovulation with conventional stimulation protocols (4). Despite the consensus of ESHRE working group on “Bologna criteria” for the definition of POR, there is no universally acceptable definition (5). Depending on different definition, POR represents 9-24% of patients undergoing ovarian stimulation for assisted reproductive program (6). The optimal stimulation protocol for poor responder patients is a therapeutic challenge. High dose gonadotropins administration, gonadotropin releasing hormone (GnRH) agonist “flare-up” regimens, natural cycle IVF, the addition of estradiol in the luteal phase, and adjunct use of different substances like growth hormone, androgenic agents, aspirin, pyridestigmine, and L-arginine have been employed (7,8). Several authors have proposed GnRH antagonist protocol (9, 10). 

The lack of initial central down-regulation in early follicular phase and adequate prevention of premature luteinizing hormone (LH) surge in late follicular phase provide GnRH antagonist protocol as a potentially proper option for poor responders (9). Significant reduction in gonadotropin dosage and stimulation period could be achieved by antagonist protocol. Nevertheless, there are no significant differences in terms of clinical pregnancy and cancellation rates between the GnRH antagonist and agonist cycles in poor responder patients (10). 

Therefore, this subset of patients might be the best to benefit from new treatment strategies that make better outcomes. The use of aromatase inhibitors in a GnRH antagonist protocol was suggested by some studies (11-13). Yarali *and colleagues *demonstrated that adjuvant therapy with letrozole could improve the response in poor responder patients (11). Meanwhile, in another study, adding letrozole to ovarian stimulation has no positive effect on the likelihood of pregnancy in poor responders (12).

Letrozole is a selective, non-steroidal third generation aromatase inhibitor. Letrozole causes a reduction in conversion of androstenedione and testosterone to estrone and estradiol by inhibiting the aromatase enzyme activity (14). According to some published studies, the decline in early follicular phase estrogen levels, and consequently decrease in negative feedback of estrogen on FSH release in hypothalamic-pituitary axis cause an increase in endogenous gonadotropin secretion and stimulation of ovarian follicular growth (14,15). 

In addition, an increase in intraovarian androgens secondary to aromatase inhibition, augments the follicular sensitivity to FSH stimulation and follicular growth (16). Letrozole has no antiestrogenic effect over the endometrium (17). These reports prompted us to hypothesize that use of letrozole as a co-treatment agent in GnRH antagonist protocol might enhance cycle outcomes.

In this prospective study, we compared GnRH-antagonist protocol involving letrozole overlapping to a standard GnRH-antagonist protocol in poor responder patients. 

## Materials and methods

**Patients**

This randomized, double-blinded, clinical trial study was conducted on infertile women with poor ovarian response based on Bologna criteria (5) referred to the IVF Unit, Department of Obstetrics and Gynecology, Yas Hospital, Tehran, Iran between March and August 2015. At least two of three features should be contemporaneously present in each patient:

1- Poor ovarian response in the previous cycle: At least one previous failed IVF/ICSI cycle with conventional long-agonist protocol and less than four mature oocytes

2- Decreased ovarian reserve: AFC < 5-7 or AMH < 1.1 ng/mL.

3- Age of participants’ partner ≥40 years old

The women with at least two episodes of poor ovarian response (≤3 oocytes with conventional stimulation protocol) after maximal stimulation were defined as POR in absence of advance age or diminished ovarian reserve. 

The exclusion criteria were as below: 1) Metabolic or endocrine disorders including hyperprolactinoma and hypo/hyperthyroidism, 2) Endometriosis, 3) History of previous surgery on ovaries, 4) Body mass index >30 kg/m^2^, and 5) Azoospermic male partner. 

There were 2-3 un-intervened cycles between the last ovarian stimulation attempt and the current study. Random Number generator version 1.0 (Segobit Software, Issaquah, WA, USA) was used to randomly assign the participants in a 1:1 ratio to either adding letrozole or placebo to GnRH-antagonist stimulation protocol. The randomization sequence was concealed using sequentially numbered, opaque, and sealed envelopes. The participants, clinicians, and the statistician were blind to allocation. Separate individuals were responsible for rating the women and random allocation.

**Treatment protocols**

All women were evaluated on the 2^nd^ day of cycle with transvaginal ultrasound (4.5-7 MHz vaginal probe, Sono line G-40, Siemens, Germany) for measuring endometrial lining and performing an antral follicle count. Base line serum FSH, LH, E2, and progesterone (P) levels were also measured in initial assessment before gonadotropin stimulation. In both groups, ovarian stimulation was started by recombinant human follicle stimulating hormone (rhFSH) (Gonal-ƒ; Merck Serono, Modugno, Italy) 225 IU subcutaneously (SC) on day 3 of the cycle. The LA group received letrozole (Letrofem®; Iran hormone, Tehran, Iran) from day 3 of the cycle, 2.5 mg orally per day for five days. *In the*PA group, participants received placebo on the same days as oral pills. Serial ultrasound examinations and evaluation of serum E_2_ levels were used to assess follicular maturation. The dosage of rhFSH was adjusted individually according to ovarian response. When follicle(s) ≥14 mm in average diameter were observed, the GnRH antagonist, cetrorelix (Cetrotide^®^, Serono International, Geneva, Switzerland) 250mg/day subcutaneously was started and continued until the day of triggering of ovulation as a variable method. 

**Oocyte retrieval**

Choriogonadotropin alfa (Ovitrelle, Merck Serono, Modugno, Italy) 250 microgram was administrated SC for triggering of ovulation when at least two follicles measuring ≥18 mm in diameter and E_2_ serum concentration ≥500 pg/mL were obtained. Failure to achieve these criteria (less than two follicles with 18 mm diameter and E_2_ <500 pg/m L) after 10-12 days stimulation resulted in cycle cancellation for inadequate response. Trans vaginal ultrasound scan (7 MHz vaginal probe, Honda HS- 2600, Honda Company, Japan)- guided oocyte retrievals were performed 34-36 hr after ovitrelle administration under general anesthesia. Intracytoplasmic sperm injection (ICSI) technique was performed for all cycles. 

**Embryo transfer**

Up to two embryos at 4- to 8- cell stage were replaced under ultrasound scan guidance by an embryo transfer catheter (Merck, Limerick, Ireland) 48-72 hours after oocytes retrieval. All patients received progesterone vaginal/rectal suppository tablets (Cyclogest, Actavis, Barnstaple, UK) 400 mg twice daily by vaginal route, which was initiated the day after retrieval for 2 weeks, and being continued for another 8 week in cases where a pregnancy was achieved. 

A serum pregnancy test (beta human chorionic gonadotropin, β-hCG) was performed 12 days after transfer. Chemical pregnancy was defined by positive β-HCG titer 12 days after embryo transfer day. The clinical pregnancy was diagnosed by visualization of a gestational sac and fetal pole with or without fetal cardiac activity on transvaginal ultrasound scan that was performed 4-5 weeks after embryo transfer. 

**The study’s outcome measures**

The main outcomes in this study were the number of oocytes retrieved, fertilization rate, implantation rate, cycle cancelation rate, and clinical pregnancy rate. The secondary outcomes were daily gonadotropin dose, duration of gonadotropin stimulation, the endometrial thickness on trigger day, peak serum E_2_ levels, and the number of embryos transferred. 

**Ethical consideration**

The Ethics Committee of Tehran University of Medical Sciences, Tehran, Iran, approved the study protocol. The written informed consents were obtained from all participants included in this study.

**Statistical analysis**

The statistical analysis was carried out using Statistical Package for the Social Sciences, version 16, SPSS Inc, Chicago, Illinois, USA (SPSS). The independent t-test and ^2^ test were used where appropriate. p<0.05 was considered statistically significant. The data presented as mean±SD. 

## Results

**Figure 1:**
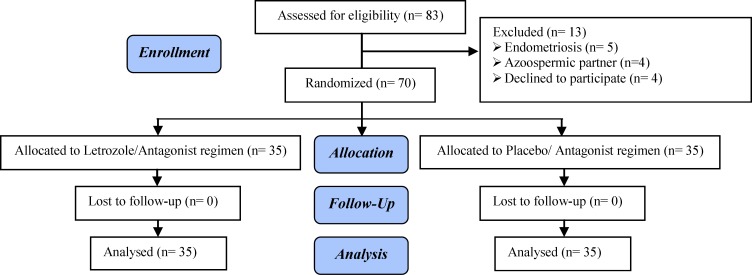
Consort flowchart. Recruitment follow-up and dropouts over the course of study

Eighty-three patients, who recognized as poor responders based on Bologna criteria, were selected. Thirteen women were excluded according to our exclusion criteria (n=9) and refusing to participate (n=4). There was no case of dropout from either group. Therefore, the data of 35 participants in each group were analyzed (Figure 1). Demographic parameters including mean female age, duration of infertility, BMI, serum AMH, basal FSH and LH levels, and AFC were similar between two groups before the initiation of stimulation protocols (Table I). Twenty-five women in LA group (71.4%) and 23 in PA group (65.7%) had at least one episode of poor ovarian response with previous conventional long-GnRH agonist protocol. The results of stimulation protocols are displayed in table II. 

There were no differences in dose and duration of gonadotropin administrated, E_2_ levels on Ovitrelle administration day, the endometrial thickness, and total number of follicles ≥16 mm as seen on ultrasonography on the day of ovitrelle administration. The number of oocytes retrieved, the number of metaphase II oocytes, the mean number of embryos transferred, and fertilization rate are comparable between two groups. Cycle outcome characteristics are displayed in table III. The total cancellation rates including poor response to stimulation protocols, failed fertilization, and arrest of embryo growth were comparable in the both groups (20% vs. 22.9%, p=0.08). There were no differences in implantation and biochemical pregnancy rates among the groups (p=0.72 and p=0.34, respectively). In LA group five clinical pregnancies (14.3%) and in PA group, four clinical pregnancies (11.3%) were recorded (p=0.12). Therefore, no statistically significant differences were noted between any of the primary and secondary outcomes in LA group and PA group (Tables II, III). No apparent side effect was reported with letrozole administration. 

**Table I:** Demographic and clinical characteristics of study participants in two groups

**Patients characteristics**	**LA group** **(n=35)**	**PA group ** **(n=35)**	**p-value**	**CI 95%**
Female age⃰ (year)	38.20± 3.41	37.9 ± 3.66	0.76	-1.97 - 1.43
Duration of infertility⃰ (year)	5.09 ± 2.04	5.77 ± 2.37	0.21	-0.37-1.74
No. of couple with primary infertility n (%)	29(85.7%)	27(77.1%)	0.09	-0.21- 1.32
BMI* (Kg/m^2^ )	23.7 ± 2.11	23.6 ± 1.90	0.81	-1.07 - 0.84
AMH level* (ng/mL )	1.69±1.17	1.55±1.05	0.52	-0.664 - 0.341
Early follicular phase FSH* (IU/L)	9.54 ±3.16	8.50 ±4.91	0.70	-7.06 - 4.98
Early follicular phase LH* (IU/L)	5.22 ±3.22	5.72 ±2.55	0.79	-3.73 - 4.74
Antral follicle count (n)	5.36 ±1.7	5.28 ±2.03	0.81	-2.79 - 2.63
Prior failed cycles (%)	71.4	65.7	0.26 ^a^	──

Groups compared using independent Student’s *t*-test, unless noted (^a^ Chi-square test was used)

BMI: Body mass index

FSH: Follicle stimulating hormone

LH = Luteinizing hormone

AMH: Anti-müllerian hormone

LA: Letrozole+Antagonist

PA= Placebo+Antagonist

* Values are expressed as mean±SDp-value≤0.05 was considered statistically significant. 95% CI: 95% confidence interval

**Table II:** Comparison of stimulation outcomes in the two study groups

**Variables **	**LA group** **(n=35)**	**PA group** **(n=35)**	**p-value**	**CI 95%**
Total gonadotropin /cycle (IU)	2475 ± 266	2625± 531	0.34	- 1.23 – 3.52
Duration of stimulation (Day)	10 ±0.70	10.2 ±0.837	0.87	- 0.93 – 1.33
Peak E_2_ level at trigger (pg/m L )	808 ± 173	693 ±199	0. 36	-3.86 – 15.84
Serum progesterone at trigger (ng/m L)	0.5 ± 0.3	0.6 ± 0.1	0.12	-0.7 - 1.1
Endometrial thickness (mm )	8.89 ±0.458	8.70±0.489	0.10	-0.41 – 0.03
Follicles ≥16 mm ( n)	3.4 ± 0.1	3.1 ± 0.7	0.08	-0.23 – 0.36
Oocyte retrieved (n)	2.80 ± 1.09	2.60±1.51	0.81	-2.12 – 1.72
Metaphase II oocytes (n)	2.03 ± 0.12	2.09 ± 0.13	0.84	-0.52 - 0.63
Good quality embryo (%)	37.1	36.8	0.42	-0.42-1.21
Embryos transferred (n)	1.2± 0.75	1.23 ± 0.74	0.82	-0.35 - 0.41
Fertilization ratea (%)	72.2	69.3	0.22	────

Values are expressed as mean±SD or percentage (%). p-value ≤ 0.05 was considered statistically significant.

Groups compared using independent Student’s *t* test unless noted (^a^ Chi-square was used). 95% CI: 95% confidence interval

E_2_= Estradiol

LA: Letrozole+Antagonist

PA= Placebo+Antagonist

**Table III:** Comparison of cycle outcomes in the two study groups

**Variables**	**LA group** **(n=35)**	**PA group** **(n=35)**	**p-value**
Total cancellation rate	20	22.9	0.08
Canceled cycle due to poor ovarian response	15.6	16.3	0.14
Canceled E.T after retrieved ^a^	4.4	6.6	0.24
Implantation rate	11.9	9.5	0.72
Biochemical pregnancy rate	25.7	20	0.34
Clinical pregnancy rate	14.3	11.4	0.12

Values are percentage (%). Groups compared using chi-square test. P-value ≤ 0.05 was considered statistically significant.

(a) Due to failed fertilization or arrest of embryo development.

LA: Letrozole+Antagonist

PA= Placebo+Antagonist

## Discussion

The present study was an endeavor to evaluate the potential role of letrozole as an adjuvant drug to improve the cycle outcomes of standard GnRH antagonist stimulation protocol in poor responder patients. The result of the current trial showed no significant difference in the number of oocytes retrieved, fertilization rate, implantation rate, cycle cancelation rate, and clinical pregnancy rate with adding letrozole to GnRH antagonist cycles in women with POR.

The introduction of GnRH-antagonist in stimulation protocols of poor responders has offered an improvement in treatment modalities by overcoming any possible negative effects of GnRH-agonist on ovaries with a limited reserve. The combination of GnRH antagonist and gonadotropins takes advantages of an initial release of endogenous gonadotropins, hyperstimulation by exogenous gonadotropins, and prevention of premature LH surge (9, 10). Some studies demonstrated that GnRH-antagonists yield a significant decrease in dosage and duration of gonadotropin administration (10, 18). Never-theless, cycle cancellation rate, implantation rate, and clinical pregnancy rate did not differ under the use of GnRH-antagonist (18). Therefore, alternative strategies should be tried to achieve better outcomes.

There are considerable evidences in the literature to support the close relationship between endogenous (serum and intra-follicular) androgen levels and early follicular growth (19-21). Androgens have the enhancing effects on follicular steroido-genesis, granulosa cells development, and ovarian responsiveness by stimulating insulin growth factor (IGF-1) and IGF-1 receptor genes expression in granulosa cells (22). Low endogenous androgen levels have been associated with impaired clinical outcomes after IVF program (23). The adjuvant use of androgens (dehydroepiandrosterone, dehydroepiandrosterone sulfate, and testosterone) in poor responders undergoing IVF treatment was accompanied by a decline in gonadotropin consumption and significant improvement in AFC, the number of oocytes, ongoing pregnancy, and live birth rates (8, 24).

Letrozole is a selective, non-steroidal aromatase inhibitor. It blocks androgen conversion to estrogen. Letrozole is a desirable drug due to its oral administration and low cost. The brief half-life (~45 hr) allows the rapid disappearing of drug and complete endometrial recovery before implantation and early embryogenesis (25). An Initial study has suggested an increase in cardiac, musculoskeletal system malformations and low birth weight in offspring of mothers who conceived with letrozole (26). Nonetheless, in several studies, the pregnancies conceived after use of letrozole for ovulation induction were associated with similar risks of spontaneous abortion and congenital anomalies compared with pregnancies achieved without ovarian stimulation. The side effects have occurred rarely in patients that were treated for ovulation induction (27, 28). 

The reduced pregnancy prospect in poor responders may be attributed to the effect of short follicular phase and low FSH receptor expression in granulosa cells (19). The letrozole-mediated decrease in serum estrogen levels and temporary enhance in intraovarian androgen concentrations cause prolongation of the follicular phase, increase in affinity of FSH receptors, preantral and antral follicles growth, and consequently enhance ovarian response to stimulation protocol (12, 19). Moreover, the reduced serum E_2_ concentration achieved with letrozole may limit the negative effect of cumulative E_2_ levels on oocyte quality and endometrial receptivity in ART cycles (15, 29). Garcia-Velasco *and colleagues *added letrozole to stimulation program of poor responder women and showed increased intrafollicular androgen concentrations and improvement in ovarian response (19). They postulated that it might be due to letrozole- induced PCO-like condition and an increment in preantral and antral follicles number (19, 22).

Sekhon *and colleagues *selected 90 women with poor ovarian response in previous GnRH-antagonist cycles and added letrozole in the early follicular phase of the subsequent cycle. They found a decline in gonadotropin requirement and the trend toward improvement in implantation and ongoing pregnancy rates in letrozole added group (30). In Goswami and colleagues study, long GnRH- agonist stimulation protocol with high dose gonadotropin (330-450 IU/day) was compared with a letrozole/low-dose gonadotropin (75 IU/day) combination regimen in poor responders. Letrozole/gonadotropin group significantly received a lower gonadotropin dosage. Clinical outcomes were comparable in both groups (12). These results were in accordance with what has been reported by various studies (19, 31-33). 

In the light of these findings, we seek to elucidate whether adding letrozole to a GnRH- antagonist stimulation protocol improves ICSI outcomes in patients who defined as “poor responder” by Bologna criteria (5). In previous studies, letrozole has been administrated in doses of 2.5, 5, and 7.5 mg per day for induction ovulation (34, 35). In comparison of these doses, there were no statistically significant differences in pregnancy rates (36, 37). Garcia-Velasco *and colleagues *showed a significant improvement in IVF outcomes in poor responders by adding 2.5 mg of letrozole to the first five days of antagonist stimulation program (19). Therefore, we chose using the lowest dose (2.5 mg) per day for five days in early follicular phase to avoid the adverse effects. Despite above-mentioned studies, we demonstrated that incorporation of low dose letrozole to a GnRH antagonist stimulation protocol could not be an effective way to improve ICSI outcomes.

We postulated that the discrepancy between our results and previous studies might be possible due to the use of different criteria for the definition of POR. We used “Bologna criteria” to define POR. In various studies, different definitions for POR and different cut-off values for ovarian reserve tests, the number of retrieved oocytes, and E_2 _levels on the day of HCG injection have been used (12, 13, 19). We did not find properly design clinical trials based on “Bologna criteria” to compare our results. Another explanation of differences between our results versus above-mentioned studies may be attributed to the effects of the two different doses of letrozole used (i.e. 2.5 vs. 5 mg) or the different starting days (32, 33).

On the other hand, the result of studies with positive findings should be assessed with caution. In Garcia-Velasco study, despite the higher number of oocytes retrieved and marked improvement in implantation rate in the letrozole-added group, no significant differences were found regarding cycle cancellation, fertilization, and pregnancy rates between the compared groups (19). In fact, there are cyclic differences in a cohort of recruitable follicles and variability in ovarian response to stimulation programs. One episode of poor response to a stimulation protocol will repeat in the second attempt, with the same protocol in only 46-62.4% of cases (38, 39). In light of this observation, the improvement in cycle outcome with adding letrozole might be related to the variability in ovarian response not to drug effect.

The findings obtained in studies with positive results have been influenced by some limitation in the search strategy. The limitations related to the methodological drawbacks (small study population**,** lack of randomization, and the different mean age of patients in the compared groups), retrospective nature, and lack of uniform definition for “poor ovarian response”, do not allow to compare the results of these trials and identify firm conclusion (13, 30, 32, 33).

In the other hand, the presumable effects of exogenous androgens on increasing intra-ovarian androgen concentration could not be extended to adjuvant letrozole. The growing follicles require several weeks to reach the antral stage. In studies with positive results, androgens have been started several days or weeks before starting gonadotropins (24, 40). In studies with the subject of the effect of adding letrozole on IVF stimulation protocols, letrozole has been administrated few days before or along with gonadotropins for a limited duration (five days) (12, 13, 31-33). Therefore, a significant change in intra-ovarian bioavailability of androgens would not achieve by letrozole. Moreover, some published studies demonstrated no statistically significant difference in terms of stimulation duration, the number of retrieved oocytes, and clinical pregnancy rates after receiving exogenous androgens (directly by androgenic agents or indirectly by aromatase inhibitors) in poor responders undergoing ART (12, 41). 

The main criticism in our study is small sample size. The relatively small population of infertile patients (9.3%) (unpublished data) who referred to our department fit Bologna criteria for POR. We cannot rule out the possibility of type II statistical errors in our results. Additionally, there is another drawback in our work with respect to study design. Preimplantation genetic study (PGS) offers improved the accuracy of embryo assessment and selection (42). This technology requires an extended culture to the blastocyte stage and trophectoderm biopsy. In this study, E.T was performed on cleavage stage before the PGS application. Thus, an increased sample size and PGS performing before E.T would be necessary to verify our findings.

## Conclusion

In conclusion, there is insufficient evidence to establish recommendation on the use of low dose letrozole as an adjuvant in ART stimulation protocols of poor responder patients. General acceptances of a uniform definition for POR and performance of well-designed prospective randomize trials with large sample size are critical to drawing the precise conclusion on the role of letrozole in stimulation protocols of poor responder patients.

## Note

***This article extracted from M.D. thesis. (Mojgan Ghalandar-Attar)***

***Registration ID in IRCT: IRCT 2014101919578N1***
